# Genomic insights into *Listeria monocytogenes* in Chengdu catering facilities: disinfectant tolerance and stress adaptation mechanisms

**DOI:** 10.3389/fmicb.2026.1750445

**Published:** 2026-05-08

**Authors:** Xueqiang Chen, Qiaoling He, Xin Lv, Shanming Lai, Jing Li, Yijun Tang, Jianjun Zhang, Biao Zhang, Xiaoqin Yu

**Affiliations:** Sichuan Institute of Food Inspection, Chengdu, Sichuan, China

**Keywords:** antimicrobial resistance, disinfectant tolerance, *Listeria monocytogenes*, stress response gene, whole-genome sequencing

## Abstract

Listeria monocytogenes poses a significant threat in food service environments due to its environmental robustness and pathogenicity. In this study, *L. monocytogenes* contamination across 24 catering facilities in Chengdu highlighted potential public health concerns, with a 6.43% (40/622) prevalence rate and meat processing areas as primary hotspots (13.48%, 19/141; cleaned meat: 25.0%, 6/24; cleaning pools: 20.83%, 5/24). Cross-contamination pathways from raw to cooking area were identified in 25% of facilities. Genomic characterization showed serotype 1/2a dominance (65.0%, 26/40), with 82.5% (33/40) of isolates belonging to serogroups frequently associated with human invasive listeriosis (1/2a, 1/2b), while ST101 (20.0%, 8/40) and ST121 (17.5%, 7/40) exhibited broad spatial dissemination. All isolates harbored disinfectant tolerance genes (*Ide/mdrL*: 100%) and stress adaptation markers (*fosX*: 100%; *cspB/D/dnaK*: 100%). Phenotypic antibiotic resistance was low (meropenem: 12.5%, 5/40; tetracycline: 2.5%, 1/40). Disinfectant efficacy testing exposed critical gaps: NaClO MICs (750–3,000 μg/mL) exceeded recommended food contact surface concentrations (240 μg/mL) by 3–12.5-fold, whereas QACs (MIC: 3.91–7.81 μg/mL) and H_2_O_2_ (MIC: 187.5–375 μg/mL) demonstrated high efficacy, with applied concentrations exceeding MICs by 64–128-fold and approximately 53–128-fold, respectively. These findings reveal a disconnect between the reduced *in vitro* susceptibility of prevalent strains to commonly used chlorine-based disinfectants and the high efficacy observed for QACs and H_2_O_2_. This integrated genomic-phenotypic approach identifies high-risk contamination nodes, highlighting its potential to inform the development of targeted control strategies for food service operations.

## Introduction

*Listeria monocytogenes* (*L*. *monocytogenes*), a Gram-positive, facultative anaerobic, rod-shaped foodborne pathogen, thrives optimally between 30 and 37°C and tolerates a broad pH range (4.6–9.5), exhibiting significant tolerance to various disinfectants (Disson et al., 2021; Manyi-Loh and Lues, 2025; Osek et al., 2022). *L. monocytogenes* has been reported in diverse foods, including animal-derived products (dairy, meat, poultry, seafood) and non-animal-derived items (fresh vegetables, fruits, cereals), frequently contaminating raw milk, raw pork, raw chicken, and other meat products (Hazards et al., 2024; Liu et al., 2025). This pathogen can become prevalent in food processing facilities when sanitation protocols are inadequate, with meat processing equipment and surfaces identified as significant contamination sources, persisting across the farm-to-fork continuum (Poswal et al., 2025; Xu Z. S. et al., 2025). These environmental surfaces, processing equipment, and utensils can serve as substrates for *L. monocytogenes* biofilm formation, where the pathogen synthesizes protective extracellular polymeric matrices (EPS) that may enhance its survival against environmental stresses (Li W. et al., 2025; Zhou et al., 2024).

*L. monocytogenes* contamination presents significant challenges to global food safety and public health (Gómez-Baltazar et al., 2025). Designated by WHO as a high-priority foodborne pathogen, this agent causes numerous annual infections (Delahoy et al., 2023). Globally, surveillance reveals increasing recognition of invasive outbreaks, exemplified by South Africa’s 2017–2018 event causing 728 illnesses and 193 deaths (Thomas et al., 2020). In China, *L. monocytogenes* likewise poses a significant annual disease burden (Li et al., 2019; Zhang et al., 2020; Zhu et al., 2023). Clinically, infection with *L. monocytogenes* can cause mild, flu-like symptoms such as fever, headache, diarrhea, or vomiting in healthy individuals, but may lead to severe, invasive disease including septicemia and meningitis in immunocompromised individuals and the elderly, contributing to its high hospitalization and mortality rates (Lin et al., 2026; Wei et al., 2026). As the final link in the food chain, catering facilities are critical control points where contamination directly threatens consumer health through ready-to-eat foods. Their high-throughput operation makes proactive surveillance essential for outbreak prevention.

Given this persistence, reducing *L. monocytogenes* contamination in food-processing facilities is a paramount priority, demanding science-based strategies to disrupt cross-contamination pathways. Studies demonstrate that the survival capacity of *L. monocytogenes* is modulated by intrinsic strain characteristics and extrinsic environmental factors—including temperature, exposure duration, nutrient availability, pH, high-pressure processing (HPP), and disinfectants (Köster et al., 2014; Wiśniewski et al., 2024; Xu Z. S. et al., 2025; Xu B. et al., 2025). Stress tolerance adaptations enable *L. monocytogenes* colonization and persistence across food chain niches, establishing persistent contamination reservoirs that ultimately facilitate human infection (Bucur et al., 2018). Although food business operators can control *L. monocytogenes* contamination by implementing measures such as robust environmental monitoring programs, ensuring hygienic conditions on food contact surfaces, and training staff on proper hygienic practices and food safety awareness, conducting strain typing analysis to further clarify the transmission pathways of *L. monocytogenes* and the factors influencing its survival is crucial for effective contamination prevention and control (Félix et al., 2023; Wu et al., 2022).

Thus, implementing a comprehensive *L. monocytogenes* surveillance program incorporating isolate subtyping is critical for identifying contamination reservoirs and resident strains to guide targeted eradication strategies (Pightling et al., 2019; Pracser et al., 2024). As the methodological gold standard, whole-genome sequencing (WGS) provides unparalleled resolution in foodborne pathogen surveillance, revolutionizing *L. monocytogenes* subtyping and outbreak beyond conventional serotyping limitations (Lüth et al., 2025; Mixão et al., 2025). The implementation of WGS enables comprehensive genomic annotation of *L. monocytogenes* isolates from catering facilities, characterizing antimicrobial resistance (AMR), antibiotic resistance genes (ARGs) and stress response gene profiles (D’Aeth et al., 2025; Pracser et al., 2024). Furthermore, this approach concurrently facilitates multi-locus sequence typing (MLST)-based serotype prediction, phylogenetic reconstruction of strain relatedness, contamination route mapping, and dynamic tracking of emergent strain phenotypes (Kapatai et al., 2016).

The pathogenicity of *L. monocytogenes* is primarily governed by its virulence factors, while antimicrobial resistance (AMR) capabilities may enhance its survival under antibiotic pressure and complicate clinical management (Li H. et al., 2025; Zhu et al., 2023). Critically, horizontal gene transfer facilitates AMR dissemination among *L. monocytogenes* strains (Goh et al., 2024). The emergence of resistant clones is further driven by antimicrobial selective pressures in human, animal, and food production environments, compounded by widespread antibiotic misuse that complicates clinical management (Sołtysiuk et al., 2025). Consequently, tracking ARGs is essential for monitoring AMR evolution, enabling preemptive outbreak detection and complementing phenotypic surveillance. Given the predominance of foodborne transmission—particularly via contaminated meat products—continuous monitoring of *L. monocytogenes* prevalence and AMR across the meat supply chain (production to retail) is imperative.

This study investigates *L. monocytogenes* contamination across diverse food production environments in Chengdu (China), encompassing raw ingredients, semi-processed and finished foods, food-contact surfaces, food handlers, and equipment. WGS was employed to annotate virulence genes (VGs) and ARGs, predict serotypes, and reconstruct phylogenies for contamination source-tracing. Antimicrobial susceptibility testing established phenotypic resistance profiles, enabling genotype-phenotype correlation analyses. By updating strain biological signatures and expanding the national *Listeria* surveillance repository, this integrated approach aims to delineate transmission pathways and provide foundational evidence for targeted intervention strategies and improved hygiene practices in the catering industry.

## Materials and methods

### Sample source and collection

Sampling was conducted across 24 high-throughput food service establishments (Sites A-X) in Chengdu, China, each serving > 1,000 patrons daily. A total of 622 specimens were collected under standardized protocols. The sampling strategy was designed to cover major functional areas within each facility. Each of the 24 facilities was visited once during the study period (between 2024–01 and 2025–01). Within each facility, sampling sites (e.g., cleaned meat surfaces, cleaning pools) were selected based on risk assessment of potential contamination points. A minimum of one sample per designated site type per facility was collected, with the total number per site varying depending on the size and layout of the facility. Environmental surfaces were sampled following ISO 18593-2018 using sterile cellulose sponge swabs; raw materials and semi-finished products adhered to Chinese National Standard GB 4789.1-2016. All *L. monocytogenes* isolates were logged in Supplementary Table 1 with complete metadata. Upon laboratory arrival, all samples were refrigerated (4°C) and analyzed within 24 h (Harakeh et al., 2009).

### Bacterial isolation and identification

Food samples (25 g aliquots) were aseptically weighed into sterile stomacher bags (Guangzhou Honghuan Trading Co., Ltd., China) containing 225 mL of sterile 1% (w/v) peptone water. Homogenization was performed using a Stomacher 400 blender (BeiJing Lab. Bio Tech Co., Ltd., China) at 230 rpm for 2 min, followed by primary enrichment at 30 ± 1°C for 24 ± 2 h (Guel-García et al., 2024). Serial 10-fold dilutions of pre-enriched cultures were prepared, with secondary enrichment at 30 ± 1°C for 24 ± 2 h. For environmental samples, swabs suspended in 10 mL of neutralizing buffer (e.g., D/E Neutralizing Broth) were aseptically transferred to 90 mL of Half-Fraser Broth (LB1) and incubated at 30 ± 1°C for 24 ± 2 h; after mixing, 0.1 mL was inoculated into 10 mL of Fraser Broth (LB2) for secondary enrichment at 30 ± 1°C for 24 ± 2 h. Selective isolation was performed by plating onto Listeria-selective CHROMaga™. Plates were incubated at 36 ± 1°C for 24–48 h. Up to five typical colonies per positive plate were streaked onto TSA-YE plates for purification (Sołtysiuk et al., 2025). Bacterial species identification was performed through full-length 16S rRNA gene sequencing (Sanger method) with subsequent confirmation using the VITEK^®^ 2 Compact automated system (bioMérieux, Marcy-l’Étoile, France). All authenticated pure cultures were cryopreserved at –80°C in tryptic soy broth with 50% (v/v) glycerol for long-term storage.

### Whole genome sequencing and bioinformatics analysis

Genomic DNA was extracted from pure cultures using the QIAamp DNA Mini Kit according to the manufacturer’s instructions. DNA concentration and quality were assessed using a NanoDrop spectrophotometer. The quality of DNA was considered acceptable when the A260/A280 ratio was between 1.8 and 2.0. Sequencing libraries were constructed using the NEBNext Ultra II DNA Library Prep Kit for Illumina. Library quality was verified using an Agilent 2100 Bioanalyzer, and concentration was quantified via qPCR. Sequencing was performed on an Illumina NovaSeq 6000 platform (PE150). Acquired ARGs were detected using ResFinder v3.2 (90% identity, 60% coverage threshold) (Gu et al., 2020; Zankari et al., 2012). Assembled genomes were submitted to the Foodborne Pathogen Traceability Network for serotype prediction, MLST, and gene annotation.

After initial quality assessment of raw sequencing data with FastQC v0.12.0, adapter trimming and read filtering were performed using Fsatp v0.23.4 to remove sequences shorter than 65 bp and low-quality reads (average Phred score Q < 30). Genomes were assembled *de novo* with Spades v3.15.4, iteratively polished for base accuracy using Pilon v1.24, and the quality of the final assemblies was evaluated with QUAST v5.2.0. High-quality assembled genomes were taxonomically classified using GTDB-Tk v2.3.2 (reference database R220). For functional annotation, open reading frames (ORFs) were predicted with Prodigal v2.6.3. The predicted protein sequences were aligned against specialized databases—the Comprehensive Antibiotic Resistance Database (CARD v3.2.8), the Virulence Factor Database (VFDB 2022), and BacMet v2.0—using Diamond v2.1.8 (BLASTP mode) with stringent thresholds (sequence identity ≥ 90%, coverage ≥ 60%). Pathogen-specific characterization was conducted using a custom local database built from the *L. monocytogenes* database on PubMLST^1^ for annotating serotype-determining and stress-resistance genes. MLST was performed with MLST v2.22.0 to assign sequence types (STs). Finally, a Minimum Spanning Tree (MST) was constructed and visualized using GrapeTree v1.5.0 to elucidate the genetic relationships among the isolates.

### Antimicrobial susceptibility testing

Antimicrobial susceptibility testing. The minimum inhibitory concentrations (MICs) of *L. monocytogenes* isolates against 12 antimicrobial agents were determined using the broth microdilution method, performed in accordance with the Clinical and Laboratory Standards Institute (CLSI) guidelines (CLSI M45-A3, 2015). The antimicrobial panel included: trimethoprim-sulfamethoxazole (TMP-SMX), meropenem (MEM), ampicillin (AMP), penicillin (PEN), erythromycin (ERY), vancomycin (VAN), ciprofloxacin (CIP), amikacin (AMK), tetracycline (TET), gentamicin (GEN), streptomycin (STR), and chloramphenicol (CHL). The microtiter plates were incubated at 35 ± 2°C for 16–20 h in ambient air. Quality control was conducted in each batch of tests using the following reference strains: Staphylococcus aureus ATCC^®^ 29213, Enterococcus faecalis ATCC^®^ 29212, and Escherichia coli ATCC^®^ 25922. The MIC results for these quality control strains fell within the acceptable ranges defined by CLSI. Interpretation of resistance was based on the breakpoints established in CLSI M100 (2023 edition).

### Disinfectant susceptibility testing

The MICs of seven commercial disinfectants against the 40 *L. monocytogenes* isolates were also determined using the microbroth dilution method. The disinfectants tested were: chlorine dioxide (ClO_2_), 10% didecyldimethylammonium bromide (DDAB), benzalkonium chloride (BZK), 12% hydrogen peroxide (H_2_O_2_), sodium dichloroisocyanurate (NaDCC), sodium hypochlorite (NaClO), and quaternary ammonium compounds (QACs). Additionally, five reference standard substances (ClO_2_, DDAB, NaDCC, BZK, NaClO) were included. All disinfectants and reference standards were confirmed to be within their stated expiration dates at the time of testing. Stock solutions were prepared fresh on the day of the experiment according to the manufacturers’ instructions. The quality control for disinfectant testing incorporated Staphylococcus aureus ATCC^®^ 25923, Escherichia coli ATCC^®^ 25922, and Listeria monocytogenes ATCC^®^ 19111 as reference strains.

### Statistical analyses

Statistical analyses were performed using SPSS Version 25.0. The three independent replicates were technical replicates, meaning that the MIC determination for each individual isolate was repeated three times. Data were expressed as mean ± standard deviation. Multivariate analysis, as mentioned in the results, was performed using logistic regression to assess the influence of facility and ST/serogroup on MIC profiles.

## Results

### Prevalence of *L. monocytogenes* in samples

Environmental and food samples were collected during processing operations. Sampling zones were stratified into six functional areas with corresponding *L. monocytogenes* detection rates: meat processing area (13.48%, 19/141), other areas (10.00%, 6/60), vegetable processing area (4.35%, 6/138), cooking area (3.68%, 6/163), serving area (3.13%, 3/96), and tableware washing and disinfection area (0.00%, 0/24) (Supplementary Table 2 and Supplementary Figure 1). Moreover, Supplementary Table 2 details *L. monocytogenes* detection frequencies across Sampling area in designated sampling position. Within the meat processing area, contamination occurred at all sampling area, with cleaned meat (CM) exhibiting the highest prevalence (25.0%, 6/24), followed by cleaning pool (CP) (20.83%, 5/24), worktop (WT), cutting board (CB), RAG, and meat mincer (MM) showed successively decreasing detection rates of 12.5, 8.33, 5.56, and 4.76% respectively. In other areas, refrigeration (RF) yielded a 13.16% (5/38) contamination rate. Within the vegetable processing area, RAG demonstrated the highest contamination (12.5%, 3/24). Among seven Sampling area in the cooking area, WT and semi-finished product (SFP) showed peak detection rates (8.33%, 2/24 each). In the serving area, *L. monocytogenes* was exclusively detected in finished product (FP, 6.25%, 3/48).

**FIGURE 1:**
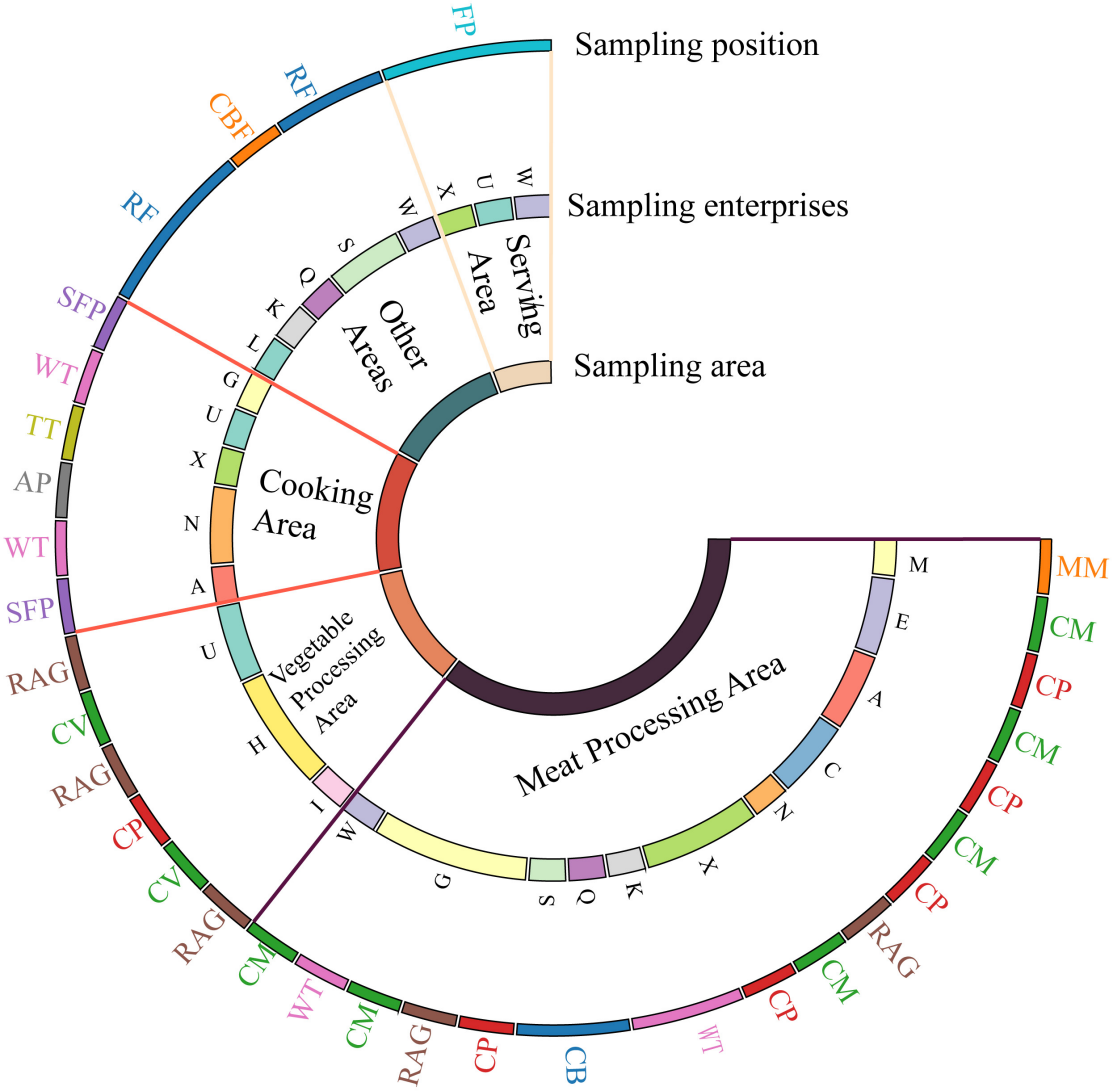
Distribution and prevalence of *L. monocytogenes* across 24 catering facilities.

Analysis of *L. monocytogenes* distribution across 24 catering facilities revealed substantial variation, with units G (19.23%, 5/26) and X (19.23%, 5/26) exhibiting the highest contamination prevalence (Figure 1 and Supplementary Figure 2). Spatial mapping confirmed pathogen presence in multiple functional zones including: unit U (serving area/cooking/vegetable processing area), W (meat processing area/serving area), X (serving/cooking/meat processing area), and N (cooking/meat processing), while units H and I showed exclusive contamination in vegetable processing area. Contamination extended to cooking and serving area in six facilities (U, W, X, N, G, L), with additional detection in meat processing area. Crucially, units G and L demonstrated co-occurrence in CM (meat processing area) and SFP (cooking area), collectively evidencing cross-contamination pathways progressing from meat processing through cooking to serving/sales areas (Hua and Zhu, 2024).

### Serotyping and MLST profiling of *L. monocytogenes* isolates

**FIGURE 2:**
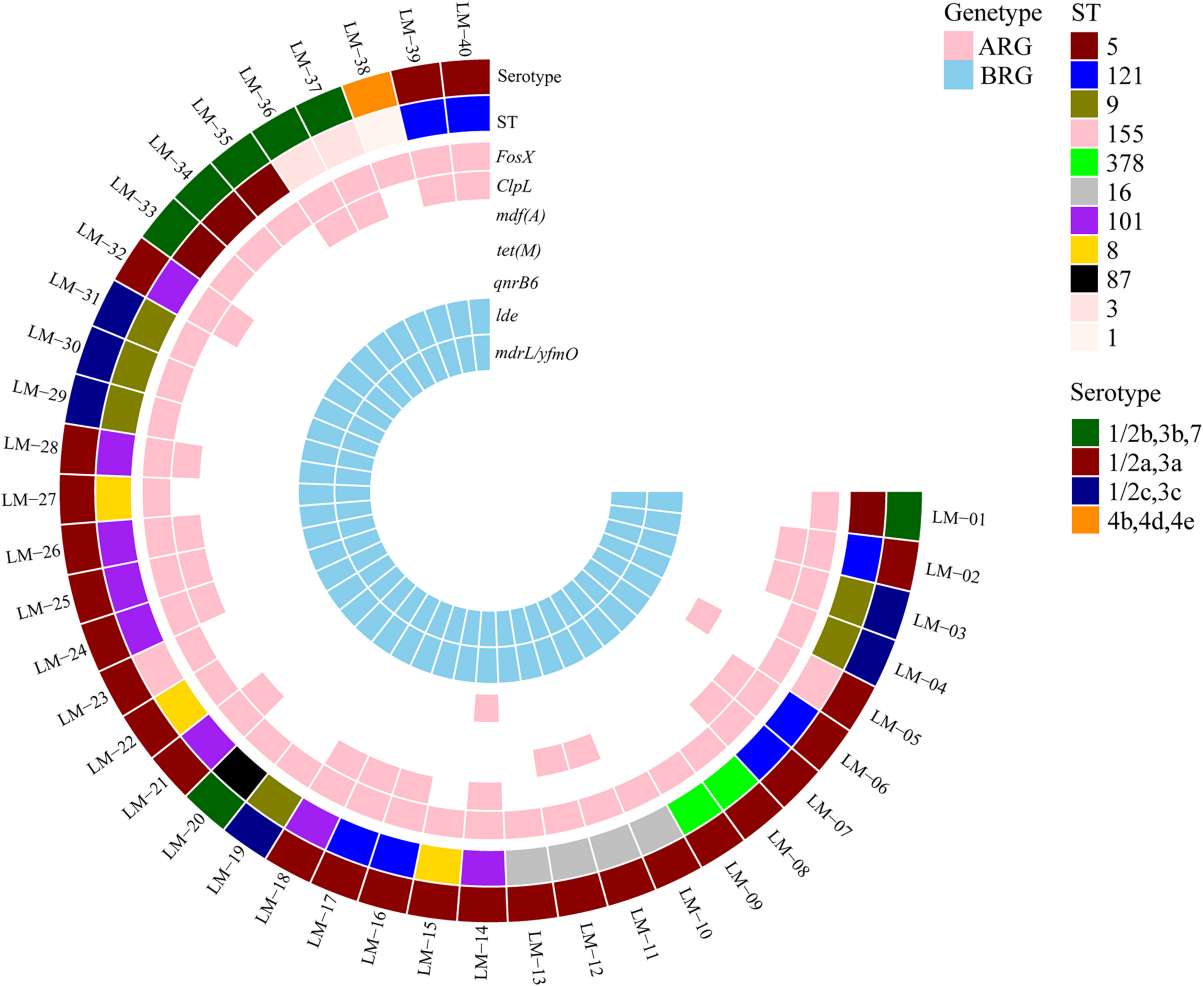
Phylogenetic relationships among 40 *L. monocytogenes* isolates based on whole-genome sequencing, showing correlation between serotypes, sequence types (STs), and genetic determinants.

In this study, WGS was performed on 40 *L. monocytogenes* isolates, with sequencing data subsequently uploaded to a national foodborne disease molecular tracing network for annotation of ARGs, serotyping, and MLST (Li et al., 2021). Integrated with isolate metadata, clustering analysis was conducted and a serogroup dendrogram constructed to elucidate phylogenetic relationships and transmission patterns across distinct ST types, serotypes, geographical sources, processing environments, and Sampling area. Results shown in Figure 2 identified serogroups 4b/4d/4e, 1/2b/3b/7, 1/2a/3a, and 1/2c/3c among the isolates, with subtype distribution as follows: 26 isolates (65.0%) were serotype 1/2a, 7 (17.5%) were 1/2b, 6 (15.0%) were 1/2c, and 1 (2.5%) was 4b. Notably, the 26 serotype 1/2a and 7 serotype 1/2b isolates exhibit invasive potential, collectively representing 82.5% of strains and indicating significant pathogenic risk.

Moreover, among 40 *L. monocytogenes* isolates, 35 strains (excluding five novel ST types) were classified into 11 distinct sequence types (STs): ST1, ST3, ST5, ST8, ST9, ST16, ST87, ST101, ST121, ST155, and ST378. As illustrated in Figure 2, ST101 (20.0%), ST121 (17.5%), and ST9 (15.0%) emerged as the predominant types, collectively representing 52.5% of isolates. Secondary prevalence was observed in ST5 (10%), ST16 (10%), ST8 (7.5%), followed by ST155, ST378, and ST3 (each 5.0%), while ST87 and ST1 demonstrated the lowest frequencies at 2.5% each.

### Distribution of antibiotic resistance and disinfectant tolerance genes in *L. monocytogenes*

Following the establishment of the population structure via MLST clustering, subsequent analysis focused on the distribution of genetic determinants associated with environmental adaptation and antimicrobial resistance. As depicted in Figure 2 and Supplementary Table 3, comprehensive antimicrobial resistance profiling identified five resistance determinants among the 40 *L. monocytogenes* isolates: *fosX*, *qnrB6*, *tet(M)*, *mdf(A)*, and *ClpL*. Universal carriage (100%) was observed for *fosX*, followed by *ClpL* (45.0%, 18/40). The *mdf(A)* determinant was present in 5.0% (2/40) of isolates, while both *tet(M)* and *qnrB6* were each detected in only a single strain (2.5%). Additionally, two disinfectant tolerance genes, *Ide* and *mdrL* (also known as *yfmO*), were universally present (100%) in all 40 *L. monocytogenes* isolates.

**FIGURE 3:**
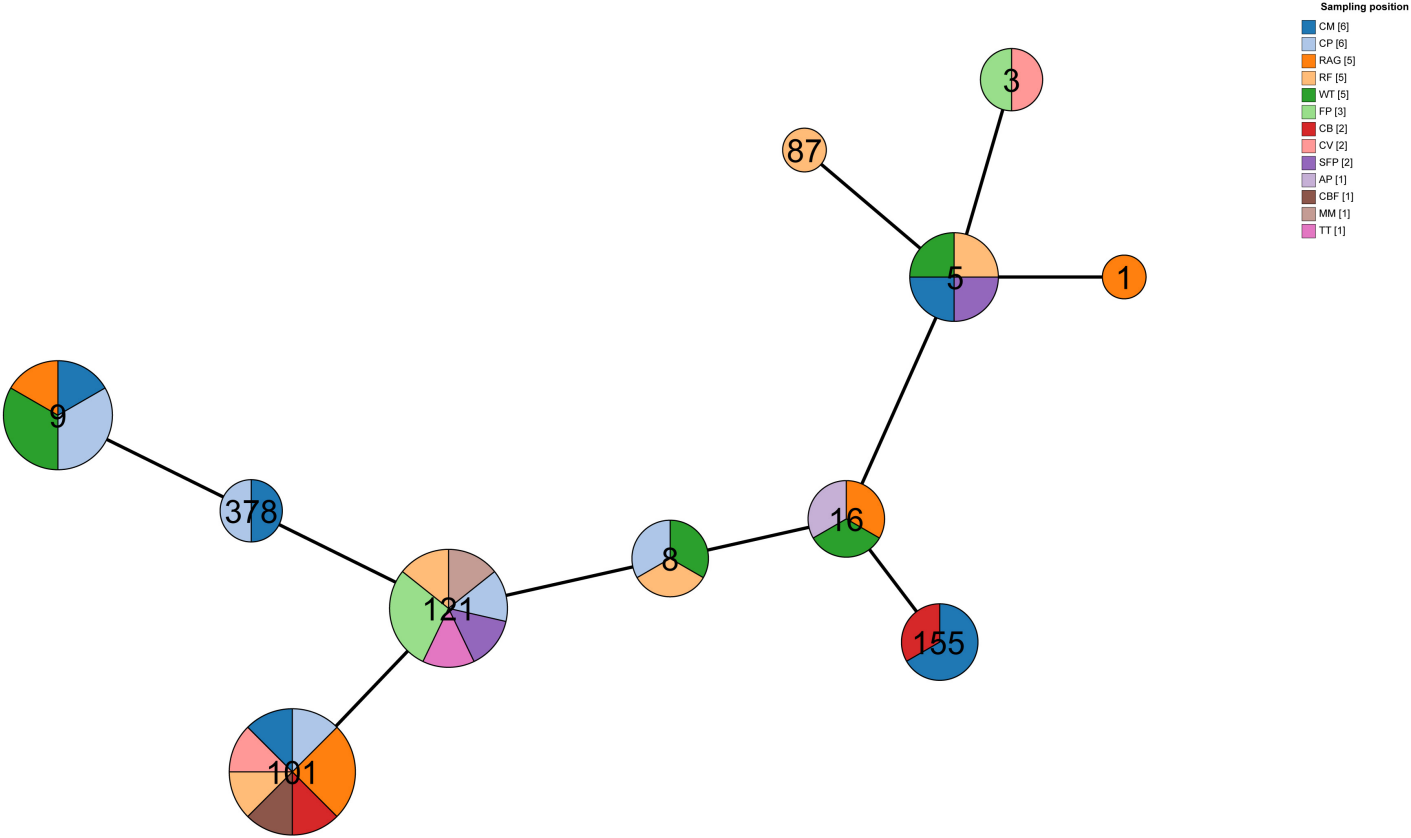
MLST profile distribution of 40 *L. monocytogenes* isolates by sampling position.

Figure 3 illustrates the MLST profile distribution of the 40 *L. monocytogenes* isolates across Sampling area. The ST101 lineage demonstrated the broadest distribution, isolated from seven distinct sites, followed by ST121, which was recovered from six different sites. Notably, ST1 was exclusively identified in RAG samples, while ST87 was found only in RF samples; all other STs were isolated from multiple sites. Furthermore, multiple STs (≥ 2) were detected at sites CM, CP, RAG, WT, FP, CB, CV, and SFP, with CM and CP exhibiting particularly high diversity, harboring up to five distinct STs each. These findings highlight the substantial diversity of *L. monocytogenes* sequence types present across the sampling locations and the varied sources of the isolates. The widespread prevalence of ST101 and ST121 across multiple food service environments is also evident.

### MIC results of *L. monocytogenes* to antibiotics

**FIGURE 4:**
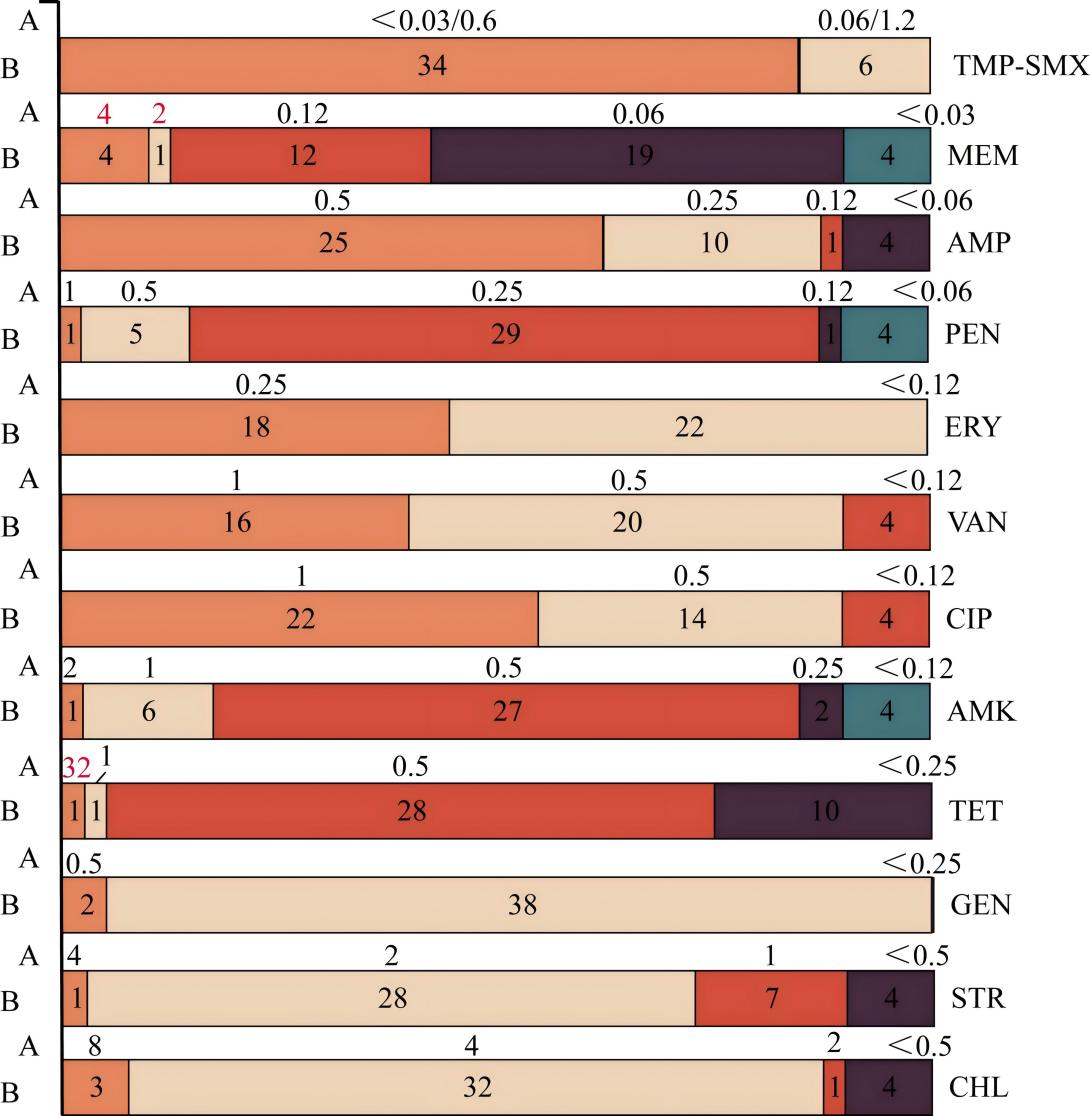
Minimum inhibitory concentration (MIC) profiles of 12 antibiotics against 40 *L. monocytogenes* isolates. (A) MIC distribution. (B) Distribution of 40 *L. monocytogenes* isolates.

The minimum inhibitory concentrations (MICs) of 40* L. monocytogenes* isolates against various antibiotics were determined by broth microdilution (Papadochristopoulos et al., 2025). Among them, 34 (85.0%) exhibited a TMP-SMX MIC of < 0.03/0.6 μg/mL, while 6 (15.0%) showed an MIC of 0.06/1.2 μg/mL (Figure 4) (47.5%), and 4 (10%) showed an MIC of < 0.03 μg/mL (4 strains, 10.0%). AMP MICs were predominantly 0.5 μg/mL (25 strains, 62.5%) and 0.25 μg/mL (10 strains, 25.0%), with additional values at 0.12 μg/mL and < 0.06 μg/mL. PEN MICs were primarily concentrated at 0.25 μg/mL (29 strains, 72.5%), with other values observed at 1 μg/mL, 0.5 μg/mL, 0.12 μg/mL, and < 0.06 μg/mL. Erythromycin susceptibility testing revealed 18 strains (45.0%) with an ERY MIC of 0.25 μg/mL and 22 strains (55.0%) with an MIC < 0.12 μg/mL. VAN MICs were most frequently 1 μg/mL (16 strains, 40.0%) or 0.5 μg/mL (20 strains, 50.0%). Similarly, CIP MICs were commonly 1 μg/mL (22 strains, 55.0%) or 0.5 μg/mL (14 strains, 35.0%). For both AMK and TET, the predominant MIC was 0.5 μg/mL, observed in 27 strains (67.5%) and 28 strains (70.0%), respectively. The majority of strains (38 strains, 95.0%) displayed a GEN MIC < 0.25 μg/mL. MICs for STR were 2 μg/mL in 28 strains (70.0%), and 32 strains (80.0%) exhibited a CHL MIC of 4 μg/mL. In summary, among the 12 antibiotics tested, the 40 *L. monocytogenes* isolates demonstrated resistance only to MEM (12.5%, 5/40) and TET (2.5%, 1/40). For CIP, 23 isolates (57.5%) exhibited MIC values at the susceptible breakpoint, corresponding to a susceptibility rate of 57.5%.

### Analysis of MIC of disinfectants against *L. monocytogenes*

Molecular characterization studies confirm persistent colonization by biocide-tolerant *L. monocytogenes* strains in industrial environments (Duze et al., 2021). The broth microdilution MIC values for seven disinfectants against 40 *L. monocytogenes* isolates from 24 food service facilities are detailed in Supplementary Table 4. The observed ranges were: ClO_2_ 1125 μg/mL, 10% DDAB 4–8 μg/mL, BZK 4–16 μg/mL, 12% H_2_O_2_ 187.5–375 μg/mL, NaDCC 500–1,000 μg/mL, NaClO 750–3,000 μg/mL, and QACs 3.91–7.81 μg/mL. NaClO exhibited the highest MICs, followed by ClO_2_, NaDCC, 12% H_2_O_2_, BZK, and 10% DDAB, with QACs demonstrating the lowest MICs. Notably, all isolates shared an identical ClO_2_ MIC (1,125 μg/mL). Strain-specific elevated MICs (2–4 × reference strain MICs) were observed: 10% DDAB (8 μg/mL in 10.0% of isolates), 12% H_2_O_2_ (375 μg/mL in 62.5%), NaDCC (1,000 μg/mL in 20.0%), NaClO (3,000 μg/mL in 15.0%), QACs (7.81 μg/mL in 32.5%), and notably BZK (16 μg/mL, 4 × reference MIC, in 10.0%). Compared to recommended use concentrations (ClO_2_ 180 μg/mL, 10% DDAB 8 μg/mL, BZK 4 μg/mL, 12% H_2_O_2_ 20,000–24,000 μg/mL, NaDCC 500 μg/mL, NaClO 240 μg/mL, QACs 500 μg/mL), only 12% H_2_O_2_ and QACs have concentrations substantially exceeding the isolate MIC ranges, indicating strong efficacy. In contrast, recommended concentrations for ClO_2_, 10% DDAB, BZK, NaDCC, and NaClO were ≤ their minimum observed MICs, suggesting potentially inadequate activity (particularly pronounced for ClO_2_ and NaClO). This reduced tolerance to BZK, associated with the *Ide* and *mdrL* determinants, suggests limited efficacy of this disinfectant in the surveyed operations. We observed operational thresholds exceeding reference MIC maxima: ClO_2_ > 269.37 μg/mL, DDAB > 8.00 μg/mL, NaDCC > 125.00 μg/mL, NaClO > 117.47 μg/mL.

### Analysis of stress-responsive genes in *L. monocytogenes*

**FIGURE 5:**
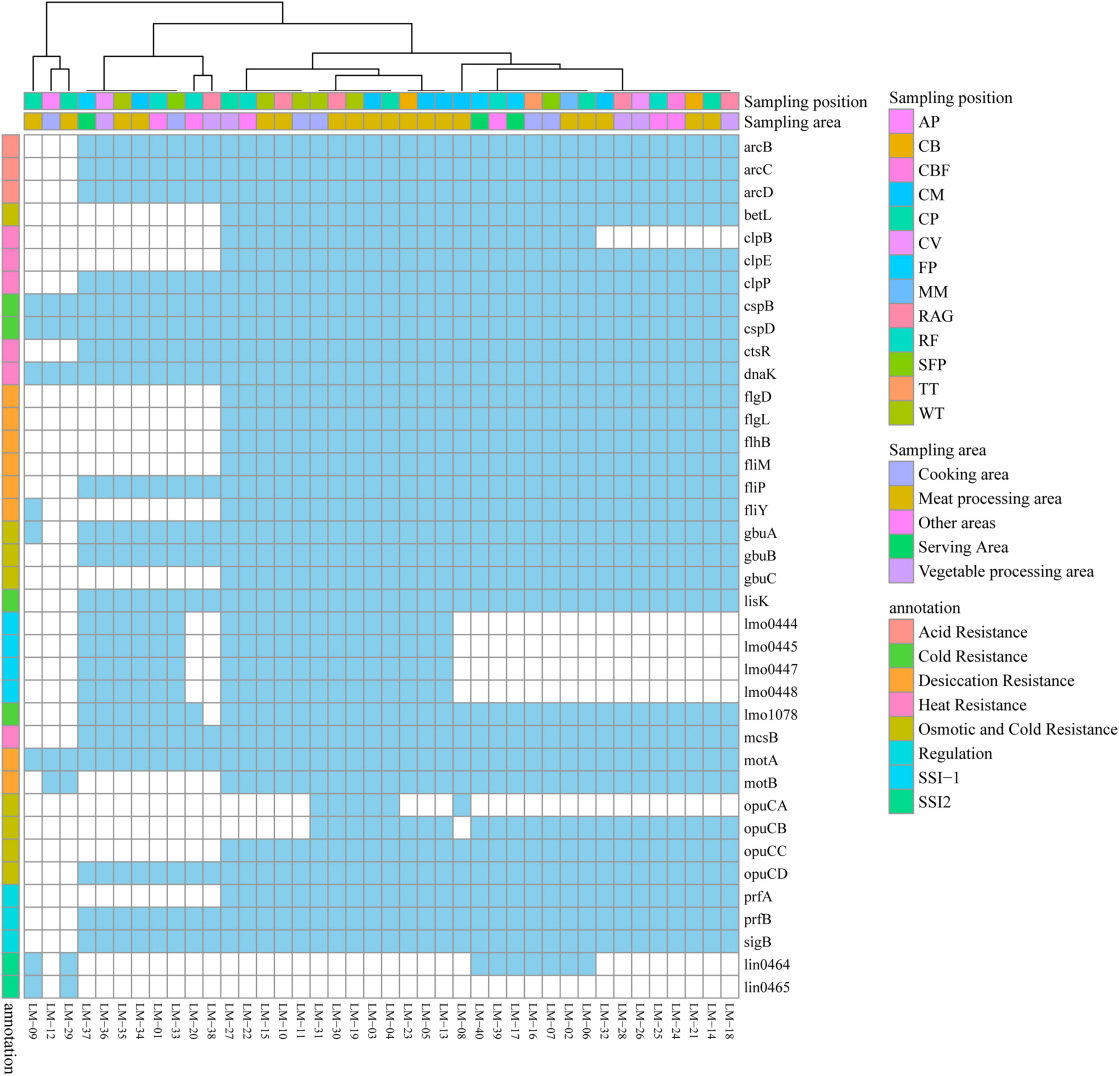
Heatmap of stress response gene distribution among 40 *L. monocytogenes* isolates, showing the universal presence of core adaptation genes (e.g., *cspB*, *cspD*, *dnaK*) and divergent profiles in potential transient contaminants.

Annotation identified 38 stress resistance genes conferring capabilities against eight distinct stresses: acid tolerance, cold adaptation, desiccation resistance, heat shock response, osmotic stress resistance, and functions associated with survival stress islands (SSI-1, SSI-2). With the exception of genes specifically linked to SSI-1 and SSI-2 functions, the majority of these stress resistance genes were ubiquitous across all 40* L. monocytogenes* isolates. Core genes essential for fundamental environmental adaptation—specifically *cspD*, *cspB*, *dnaK*, and *motA*—were universally present (100%) in the collection. This core repertoire confers crucial survival advantages under extreme conditions, including cold, heat, and desiccation. Notably, isolates highlighted within the red boxes (Figure 5), particularly the three leftmost strains, exhibited a significantly reduced complement of stress resistance genes (harboring only 5–8 genes), suggesting potential genetic divergence in their origin compared to other isolates. Collectively, the analysis of stress resistance genes, combined with antimicrobial resistance (Figure 2) and MLST profiling (Figure 3), indicates substantial diversity in the sources of these 40 *L. monocytogenes* isolates. While the prevalent possession of diverse stress resistance genes provides a significant survival advantage, the observed susceptibility profiles suggest that rational application regimens combining specific antibiotics and disinfectants may effectively control *L. monocytogenes* within food service environments. Consequently, vigilant control measures targeting all stages of food service operations are essential to prevent the dissemination of these strains.

## Discussion

The isolation of *L. monocytogenes* from 6.43% (40/622) of environmental and food samples across Chengdu’s catering facilities indicates significant contamination pressure within Chinese food service environments. This prevalence exhibits substantial spatial heterogeneity, revealing critical vulnerabilities in control points. Consistent with our stratified sampling (Supplementary Figure 1 and Supplementary Table 2), meat processing areas emerged as contamination epicenters (13.48% prevalence), with alarmingly high rates observed at CM (25.0%, 6/24) and CP (20.83%, 5/24). This corroborates international studies identifying meat processing areas as primary amplification sites for *L. monocytogenes*, facilitated by biofilm-conducive equipment surfaces, protein-rich residues, and temperature fluctuations (Ohman et al., 2024). Figure 1 and Supplementary Figure 2 identified cross-contamination vectors within six facilities (U, W, X, N, G, L). Critically, the co-occurrence of identical strains in CM (meat processing area) and SFP (cooking area) in units G and L suggests pathogen translocation from raw to partially processed items. This could implicate either failures in critical thermal processes or worker-mediated transfer via contaminated utensils/gloves, highlighting areas that warrant reassessment of Hazard Analysis Critical Control Point (HACCP) protocols (Pniewski et al., 2023). Beyond processing zones, persistent reservoirs were identified in RF (13.16% contamination, 5/38), CP, and RAG, consistent with this psychrotroph’s cold-chain adaptability. The convergence of cold-enhanced adhesion, condensation-mediated surface transfer, and stress-induced adaptations may contribute to self-reinforcing contamination cycles that challenge standard sanitation protocols (Li H. et al., 2025; Pracser et al., 2024).

Our study delineates a *L. monocytogenes* population within food service environments characterized by significant genetic diversity and adaptive traits. Serotype 1/2a predominated (65.0%, 26/40), consistent with Chinese environmental and food isolates (Shi et al., 2025), but contrasting with the clinical serotype 4b dominance in Europe and North America (Maury et al., 2016). Critically, 82.5% (33/40) of isolates belong to serogroups (1/2a, 1/2b) that are frequently associated with human invasive listeriosis, underscoring a potential public health risk. The near-universal presence of disinfectant tolerance genes *Ide* and *mdrL* (100% of isolates) (Figure 2 and Supplementary Table 3), coupled with the high prevalence of stress adaptation genes (*fosX*: 100%; *ClpL*: 45.0%), provides a genetic background conducive to survival in sanitized environments. It is important to note that gene presence indicates potential, and persistence typically requires repeated isolation of the same strain over time from a location, which was not assessed in this cross-sectional study. The dominance of serotype 1/2a may reflect inherent environmental fitness and adaptation to common food processing stresses. The universal carriage of *mdrL*, associated with tolerance to BZK and QACs, aligns with reports that Chinese 1/2a strains can harbor plasmids conferring additional tolerance and heavy metal resistance (Kremer et al., 2017), suggesting possible plasmid-mediated advantages in certain environments. While acquired antimicrobial resistance genes (*qnrB6*, *tet(M)*, *mdf(A)*) were rare (each ≤ 5.0%), their detection warrants ongoing surveillance.

MLST analysis identified ST101 (20.0%), ST121 (17.5%), and ST9 (15.0%) as the dominant lineages (Figures 2, 3), a distribution consistent with environmental *L. monocytogenes* strains reported in Eastern China (Zhang et al., 2020). ST101 and ST121 exhibited the broadest spatial distribution, isolated from seven and six distinct sampling sites, respectively (Figure 3), underscoring their successful adaptation across diverse niches within the facilities. Substantial genetic diversity was evident, with sites CM and CP harboring up to five distinct STs. The detection of sequence types (STs) associated with clinical outbreaks (e.g., ST3, ST8, ST87) suggests potential links between these production environments and human infection routes. Of note, ST87, exclusively isolated from RF samples, has been reported to form biofilms with enhanced tolerance to chlorine-based sanitizers (Li et al., 2019; Mao et al., 2023). A key observation was the strong concordance between ST and serotype (e.g., all ST101 were 1/2a). This linkage suggests evolutionary selection for specific surface antigens that may be advantageous for survival in these environments.

AMR profiling (Figures 2, 4 and Supplementary Table 3) revealed no phenotypic resistance to first-line therapies PEN or AMP. Resistance among the 40 isolates was observed only to meropenem (MEM, 12.5%, 5/40) and tetracycline (TET, 2.5%, 1/40). The *tet(M)* gene was detected in the single tetracycline-resistant isolate. Universal carriage (100%) of *fosX* and the disinfectant tolerance genes *Ide* and *mdrL* (*yfmO*) was confirmed (Supplementary Table 3). While similar MIC profiles were observed among isolates from the same facility, multivariate analysis suggested that facility-wide factors, rather than specific micro-environments, were associated with these profiles. Meat processing areas, with their organic residues, may facilitate the survival and potential transfer of resistance determinants (Duze et al., 2021).

Disinfectant efficacy assessment (Supplementary Table 4) revealed critical concerns regarding commonly used agents. NaClO MICs (750–3,000 μg/mL) exceeded recommended food contact surface concentrations (240 μg/mL) by 3–12.5-fold. ClO_2_ MICs (1,125 μg/mL) were six-fold higher than typical operational levels (180 μg/mL). The universal presence of *Ide* and *mdrL* provides a mechanistic basis for this observed tolerance, with *mdrL* encoding an efflux pump and *Ide* conferring degradation capability for certain disinfectants (Hazards et al., 2024). In contrast, QACs (MIC = 3.91–7.81 μg/mL) and hydrogen peroxide (H_2_O_2_, MIC = 187.5–375 μg/mL) demonstrated high efficacy, with their in-use concentrations far exceeding the MICs of the isolates. This presents a practical disconnect: while chlorine-based sanitizers are widely used, the isolates exhibited reduced susceptibility to them, whereas less commonly used QACs and H_2_O_2_ showed superior potential efficacy *in vitro*.

The molecular basis for environmental survival is further supported by the common carriage of stress response genes. Universally present core chaperones (*cspB*, *cspD*, *dnaK*) are crucial for coping with cold, heat, and oxidative stresses (Ogunleye et al., 2025; Schärer et al., 2013). The widespread distribution of ST121, for instance, may be partly attributed to such robust stress response systems (Rajalingam and Van Haute, 2025). A few genetic outliers carrying fewer stress genes (Figure 5) might represent transient contaminants, contrasting with strains better equipped for persistence. Furthermore, stress responses can influence tolerance profiles; for example, cold stress has been shown to upregulate *mdrL*, potentially enhancing disinfectant and antibiotic efflux simultaneously (Conficoni et al., 2016).

This integrative analysis highlights interconnected risk in the studied facilities: potentially virulent clones colonize various surfaces, disinfectant tolerance genes may compromise sanitation, and stress tolerance enhances survival. The disinfectant efficacy assessment revealed a critical finding: NaClO and ClO_2_ MICs (750–3,000 μg/mL and 1,125 μg/mL, respectively) substantially exceeded recommended food contact surface concentrations (240 and 180 μg/mL), whereas QACs (MIC: 3.91–7.81 μg/mL) and H_2_O_2_ (MIC: 187.5–375 μg/mL) demonstrated high *in vitro* efficacy, with their applied concentrations far exceeding the MICs of the isolates. The universal presence of the disinfectant tolerance genes Ide and mdrL provides a plausible genetic basis for this observed reduced susceptibility to certain agents, particularly chlorine-based ones. However, it must be emphasized that these MIC values, obtained in broth culture, serve as preliminary laboratory observations. They may not directly predict sanitizer performance on environmental surfaces where factors such as biofilm formation (e.g., as reported for ST87 in RF samples), organic load, and contact time critically influence outcomes. Therefore, these *in vitro* data highlight an important area for further investigation rather than providing definitive applied guidance. Future studies should focus on validating disinfectant efficacy against resident strains, including prevalent ST101 and ST121, under conditions that mimic the complex food processing environment, such as on surface-attached biofilms and in the presence of organic matter.

## Conclusion

This study establishes a correlation between the universal presence of disinfectant tolerance genes (*Ide*/*mdrL*) in *L. monocytogenes* isolates and their phenotypically confirmed reduced *in vitro* susceptibility to chlorine-based agents. The MICs for sodium hypochlorite NaClO and ClO_2_ exceeded recommended use concentrations by 3–12.5- and 6.2-fold, respectively, whereas the applied concentrations of QACs and H_2_O_2_ substantially exceeded their MIC ranges, demonstrating high potential efficacy *in vitro*. This disparity underscores the value of integrated genomic-phenotypic surveillance but also clarifies a critical limitation: broth microdilution MICs, while indicative, are preliminary observations that may not reliably predict sanitizer performance on environmental surfaces where factors such as biofilm formation (e.g., as noted for ST87 in RF samples), organic load, and contact time are decisive. Consequently, these findings are best positioned to direct future investigative priorities rather than to prescribe immediate practice. Consequently, these findings direct future investigative priorities toward validating disinfectant efficacy against *L. monocytogenes* biofilms under conditions simulating actual sanitation challenges, which is essential for disrupting persistent contamination cycles driven by resilient, widely disseminated clones such as ST101 and ST121.

## Data Availability

The whole-genome sequencing data from the 40 *L. monocytogenes* isolates presented in the study are deposited in the China National GeneBank DataBase (CNGBdb) repository, accession number CNP0009287.

## Footnotes

1
https://bigsdb.pasteur.fr/listeria
