# Supplementary material for: Genomic insights into Listeria monocytogenes in Chengdu catering facilities: disinfectant tolerance and stress adaptation mechanisms
Source: Front Microbiol. 2026 May 8;17:1750445. doi: 10.3389/fmicb.2026.1750445 (PMC13194457; doi:10.3389/fmicb.2026.1750445)
Supplement: Supplementary file 1 [file Data_Sheet_1.docx]

Supplementary Figures and Tables

**Supplementary Table 1**. Quality control strain MIC acceptance range (µg/ml)

| Antibiotic | | MIC Acceptance Range(µg/ml) | | |
| --- | --- | --- | --- | --- |
|  |  | ATCC 25922 | ATCC 29213 | ATCC 29212 |
| Trimethoprim/Sulfamethoxazole | TMP-SMX | ≤0.5/9.5 | — | — |
| Meropenem | MEM | 0.008-0.06 | — | — |
| Ampicillin | AMP | 2-8 | — | — |
| Penicillin | PEN | — | 0.25-2 | 1-4 |
| Erythromycin | ERY | — | 0.25-1 | 1-4 |
| Vancomycin | VAN | — | 0.5-2 | 1-4 |
| Amikacin | AMK | 0.5-4 | — | — |
| Ciprofloxacin | CIP | — | — | 0.25-2 |
| Tetracycline | TET | 0.5-2 | — | — |
| Gentamicin | GEN | 0.25-1 | — | — |
| Streptomycin | STR | — | — | — |
| Chloramphenicol | CHL | 2-8 | — | — |

Note 1: "—" indicates not applicable.

**Supplementary Table 2.** *L. monocytogene*s prevalence across processing areas in Chengdu catering facilities (n=24)

| Processing area | Sampling sites | Sample quantity | Positive sample | Detection rate |
| --- | --- | --- | --- | --- |
| Meat processing areas | Cleaned meat (CM) | 24 | 6 | 25.00% |
|  | Cleaning pool (CP) | 24 | 5 | 20.83% |
|  | Worktop (WT) | 24 | 3 | 12.50% |
|  | Cutting board (CB) | 24 | 2 | 8.33% |
|  | RAG | 24 | 2 | 8.33% |
|  | Meat mincer (MM) | 21 | 1 | 4.76% |
| Other areas | Cutting board for cooked food (CBF) | 22 | 1 | 4.55% |
|  | Refrigeration (RF) | 38 | 5 | 13.16% |
| Vegetable processing area | Cleaned vegetable (CV) | 24 | 2 | 8.33% |
|  | Cleaning pool (CP) | 24 | 1 | 4.17% |
|  | Worktop (WT) | 24 | 0 | 0.00% |
|  | Cutting board (CB) | 24 | 0 | 0.00% |
|  | RAG | 24 | 3 | 12.50% |
|  | Vegetable cutter (VC) | 18 | 0 | 0.00% |
| Cooking area | Worktop (WT) | 24 | 2 | 8.33% |
|  | RAG | 24 | 0 | 0.00% |
|  | Transfer Trolley (TT) | 19 | 1 | 5.26% |
|  | Cooked food basin (CFB) | 24 | 0 | 0.00% |
|  | Chef | 24 | 0 | 0.00% |
|  | Kitchen apron (KA) | 24 | 1 | 4.17% |
|  | Semi-finished product (SFP) | 24 | 2 | 8.33% |
| Serving area | Worktop (WT) | 24 | 0 | 0.00% |
|  | RAG | 24 | 0 | 0.00% |
|  | Finished product (FP) | 48 | 3 | 6.25% |
| Tableware washing and disinfection area | Tableware (TW) | 24 | 0 | 0.00% |

**Supplementary Table 3.** Distribution of resistance genes

| Antibiotic Resistance Gene | | | Disinfectant Tolerance Gene | | |
| --- | --- | --- | --- | --- | --- |
| Gene | Number of strains | Prevalence | Gene | Number of strains | Prevalence |
| *FosX* | 40 | 100.00% | *lde* | 40 | 100.00% |
| *ClpL* | 18 | 45.00% | *mdrL/yfmO* | 40 | 100.00% |
| *Mdf(A)* | 2 | 5.00% | — | — | — |
| *Tet(M)* | 1 | 2.50% | — | — | — |
| *qnrB6* | 1 | 2.50% | — | — | — |

Note 1: "—" indicates not applicable.

**Supplementary Table 4. MIC Results of Disinfectant Products**

| Disinfectant | Recommended Disinfectant Concentration（µg/mL） | MIC（µg/mL） | | | |
| --- | --- | --- | --- | --- | --- |
|  |  | Standard Strain | Isolate | | |
| ClO_2_(Product) | 180 | 1125 | 1125（40） | — | — |
| 10%DDAB | 8 | 4 | 4（36） | 8（4） | — |
| BZK | 4 | 4 | 4（36） | 16（4） | — |
| 12%H2O2 | 20000-24000 | 187.5 | 187.5（15） | 375（25） | — |
| NaDCC | 500 | 500 | 500（32） | 1000（8） | — |
| NaClO | 240 | 1500 | 750（1） | 1500（33） | 3000（6） |
| QACs | 500 | 3.91 | 3.91（27） | 7.81（13） | — |

Note 1: Numbers in parentheses represent the count of Listeria monocytogenes at this MIC; "—" indicates not applicable.


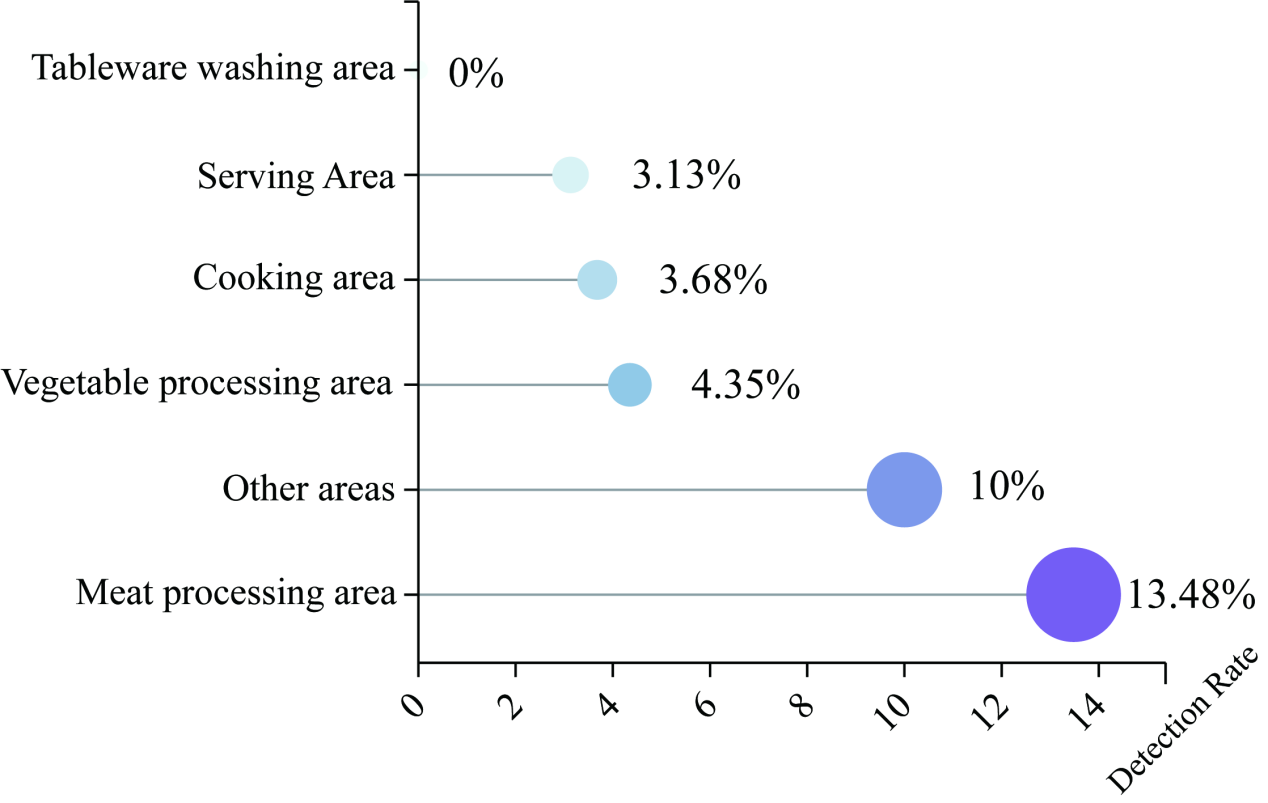


**Supplementary Figure 1.** Detection rate of *L. monocytogenes* in different sampling area.


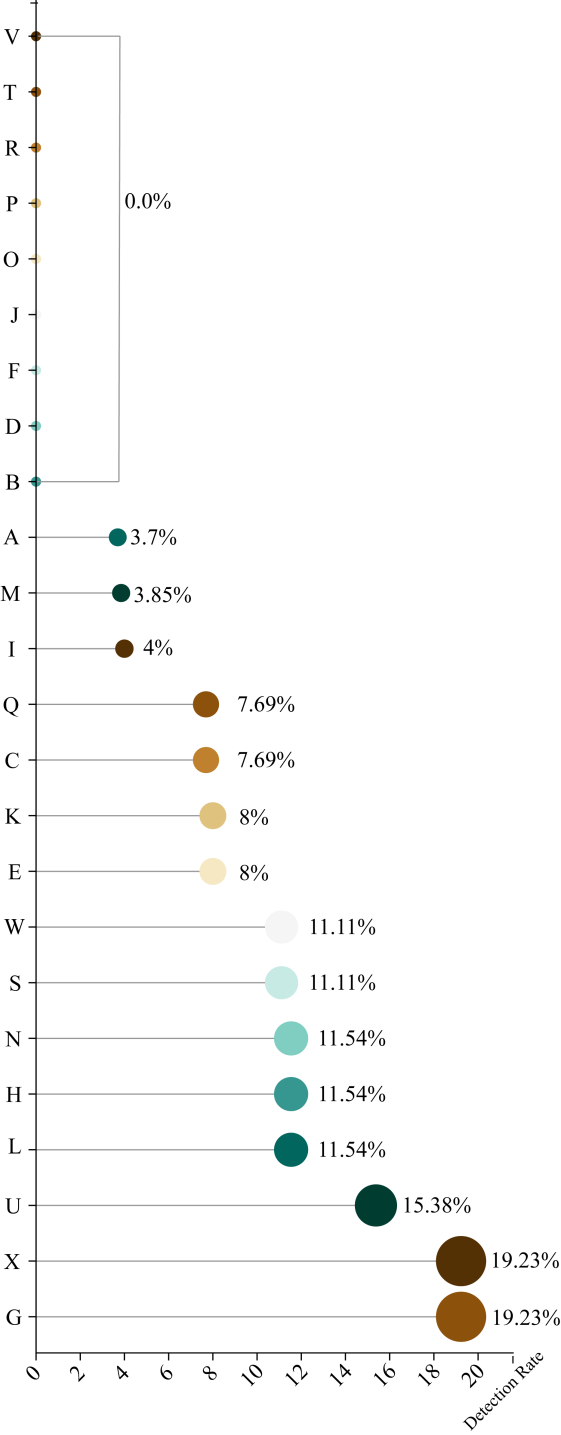


**Supplementary Figure 2.** Detection rate of *L. monocytogenes* in different catering establishments.
